# Natural History of HPV Infection across the Lifespan: Role of Viral Latency

**DOI:** 10.3390/v9100267

**Published:** 2017-09-21

**Authors:** Patti E. Gravitt, Rachel L. Winer

**Affiliations:** 1 Department of Global Health, George Washington University Milken Institute School of Public Health, Washington, DC 20052, USA; 2 Department of Epidemiology, University of Washington School of Public Health, Seattle, WA 98195, USA; rlw@uw.edu

**Keywords:** papillomavirus, latency, cervical cancer

## Abstract

Large-scale epidemiologic studies have been invaluable for elaboration of the causal relationship between persistent detection of genital human papillomavirus (HPV) infection and the development of invasive cervical cancer. However, these studies provide limited data to adequately inform models of the individual-level natural history of HPV infection over the course of a lifetime, and particularly ignore the biological distinction between HPV-negative tests and lack of infection (i.e., the possibility of latent, undetectable HPV infection). Using data from more recent epidemiological studies, this review proposes an alternative model of the natural history of genital HPV across the life span. We argue that a more complete elucidation of the age-specific probabilities of the alternative transitions is highly relevant with the expanded use of HPV testing in cervical cancer screening. With routine HPV testing in cervical cancer screening, women commonly transition in and out of HPV detectability, raising concerns for the patient and the provider regarding the source of the positive test result, its prognosis, and effective strategies to prevent future recurrence. Alternative study designs and analytic frameworks are proposed to better understand the frequency and determinants of these transition pathways.

## 1. Introduction

Prospective epidemiologic studies conducted in the late 1990s and 2000s established the temporal association between exposure to high-risk (HR) human papillomavirus (HPV) and the subsequent development of cervical intraepithelial neoplasia (CIN) and cervical cancer. These data, combined with strong biological plausibility derived from the basic sciences, led to acceptance of HR-HPV as a necessary, but insufficient cause of nearly 100% of cervical cancers [1]. Not only did these data consistently fulfill the Bradford–Hill criteria for causality, but the well-accepted temporal pathway from HPV infection to invasive cervical cancer (ICC) that was derived from these studies has also translated into important changes to cervical cancer screening guidelines worldwide, increasing both the impact and cost effectiveness of secondary cervical cancer prevention. 

Because these studies collected data on HPV repeatedly over time, typically once every 4–6 months, a natural analytic extension was to model the HPV measures as outcomes, rather than exposures, to establish incidence and clearance rates—the basic natural history parameters of the infection. These incidence and clearance estimates have formed the basis for mathematical models of HPV infection, used in health policy analyses to predict the impact of interventions such as HPV vaccination and screening. In this review, we reflect on the accuracy of these estimates in representing the natural history of viral infection within an individual over the course of the life span by reviewing the data through the lens of an infectious disease epidemiologist. We further highlight the clinical and public health relevance for enhancing our understanding of the within-woman HPV infection natural history across the life span. 

## 2. Overview of Well-Established Aspects of HPV Natural History

**Figure 1:**
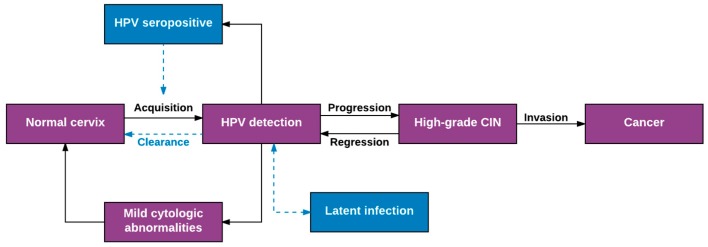
Schematic model of the population-level natural history of human papillomavirus infection and cervical cancer. Purple boxes indicate well-accepted natural history model parameters; blue boxes represent uncertainties.

Figure 1 represents the current paradigm of HPV natural history from infection to cervical cancer, and highlights several uncertainties in the interpretation of natural history estimates derived from the original prospective studies of HPV and cervical cancer. In this conceptual model, HPV infections are acquired via sexual exposures, with newly sexually active adolescent and young adult women at highest risk of acquisition [2]. During productive HPV infection, low-grade cervical abnormalities may be clinically detectable in screening (e.g., low grade squamous intraepithelial lesions (LSIL) or CIN grade 1 (CIN1)), but are usually transient and resolve without intervention within 1–2 years [3]. The majority (~90%) of newly acquired HPV infections similarly become undetectable within 1–2 years [3], a phenomenon routinely described as “viral clearance,” but which may also represent immune control below detectable levels or viral latency [4,5,6]. A detectable immune response is generated approximately 60% of the time [7], evidenced by the presence of serum antibodies specific to the HPV type causing infection, with uncertain ability to provide immunity against re-infection [8]. A minority of HPV infections are persistently detected beyond 12 months, increasing the risk of carcinogenic progression to cervical pre-cancer (high grade squamous intraepithelial lesions (HSIL) or CIN grade 2 or 2 (CIN2/3) and potentially cancer if untreated [3].

## 3. Expanded View of HPV Natural History

**Figure 2:**
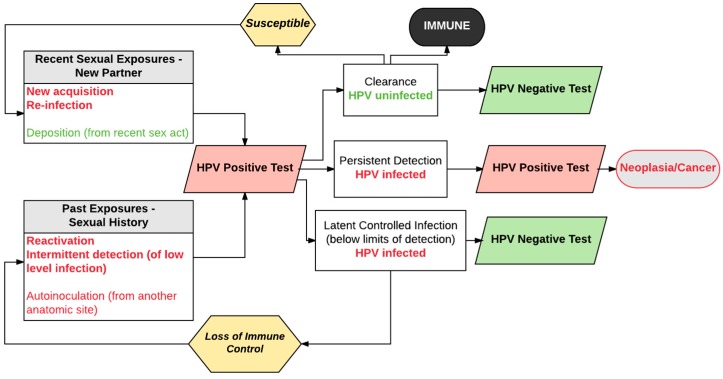
Schematic model of individual-level natural history of female genital HPV infection across the life span. The model assumes two pathways to type-specific HPV positivity after an HPV-negative test result—new acquisition or reinfection due to current sexual activity, or reactivation/recurrent detection of controlled, latent HPV infection. Red boxes indicate positive HPV molecular test results and green boxes indicate negative HPV molecular test results. Colored fonts represent the true underlying infectious status independent of concurrent molecular test results from exfoliated samples; red = HPV infection, green = HPV uninfected.

The paradigm in Figure 1 is based on our understanding of the population-level natural history of HPV over the course of 5–10 years (the typical duration of prospective natural history studies). Because of the practical limitations prohibiting longer duration studies, extrapolating this population-level model to the within-woman natural history of HPV infections over an entire life span requires several explicit assumptions. In most applications of HPV “incidence and clearance” estimates derived from this model, two critical assumptions prevail: (1) new HPV detection reflects recent acquisition either as a new infection or a re-infection; and (2) loss of HPV detection reflects viral clearance, or eradication. To more fully explore the validity of these assumptions and evaluate the evidence to support them, we have elaborated a more nuanced natural history of an HPV infection within an individual woman (Figure 2). In our model, we posit that each HPV infection may follow a number of non-linear, non-mutually exclusive pathways over a woman’s life span. Specifically, new HPV detection can result not only from a recent sexual acquisition or re-infection, but also from recurrent detection of a controlled or latent infection [9], auto-inoculation from other epithelial sites (e.g., anus) [10], or transient deposition of viral nucleic acid from a recent sex act [11]. Similarly, loss of HPV detection (aka clearance) may reflect viral eradication with or without acquired immunity against re-infection or viral control below limits of detection (aka viral latency) [12]. 

We emphasize that the short duration of typical HPV natural history studies (usually no more than 4 years of follow-up) and the infrequent sampling to measure HPV outcomes (most often only once every 4–6 months), has limited our ability to observe the full spectrum of natural history infection transitions depicted in Figure 2. A more complete elucidation of the age-specific probabilities of the alternative transitions is becoming more relevant with the expanded use of HPV testing in cervical cancer screening. Women are receiving HPV test results over a decade or more of screening, and even in the context of infrequent screening intervals (3–5 years), transitioning in and out of HPV detectability is increasingly more common, raising concerns for the patient and the provider regarding the source of the positive test result and its prognosis. While definitive answers are elusive, alternative study designs and analytic frameworks provide critical evidence to understand the frequency and determinants of these transitions. 

## 4. Evidence Supporting Re-Detection as an Important Transition from Negative to Positive HPV Tests

A key uncertainty in the natural history of HPV infection within an individual is whether an HPV infection that becomes undetectable on repeat testing has truly cleared, or whether the virus persists at low, undetectable levels or has entered a latent state. While distinctions between the latter two scenarios are controversial, studies suggest that re-detection of the same HPV type is relatively common, occurring in at least 10–20% of women observed to have “cleared” the virus [8]. Furthermore, convincing data from multiple studies of immune compromised, sexually abstinent [9,13], older, less sexually active populations [4,14], and adolescents with long-term intensive follow-up [5] support the phenomena of immunologically controlled re-detection or reactivation of a previously acquired type-specific HPV infection. The human observational data is largely consistent with animal models of papillomavirus latency, which have quite elegantly shown latent detection of low copy papillomavirus DNA with minimal gene expression in basal cells following clinical resolution of initial viral infection [15]. Upon iatrogenic immunosuppression, papillomavirus copy number increased in the epithelium of the latently infected animals, though no recurrence of warts (the consequence of initial infection) was observed [16].

## 5. New Acquisition versus Re-Detection of Prior Infection: Evidence from Studies of Mid-Adult Women

The likelihood that detectable HPV is due to new acquisition is high in newly sexually active young women [2], and decreases with age [4,14,17,18]. Although sex with new partners remains a risk factor for new infection, rates of acquiring new partners decline with age [19,20,21] and the ratio of detectable HPV that is attributable to prior versus new infection increases. These concepts are supported by data from three recent U.S. cohort studies in mid-adult women, including women aged 35–60 years in Baltimore, Maryland [14], women aged 30–50 years in Seattle, Washington [4], and female online daters aged 25–65 years recruited from various U.S. cities [17]. These studies all used repeat HPV DNA testing and sexual behavior data to estimate risks of incident HPV detection associated with recent versus past sexual exposures [4,14,17], with the Seattle study adding baseline HPV serology testing as a biomarker of prior infection [4]. Although HPV serology is an imperfect marker of prior infection (due to limited assay sensitivity [22] and the fact that antibody responses to natural infection are neither uniformly detected [7,23,24,25] nor lifelong [7,23]), the addition of serology data to analyses of HPV DNA genotyping and sexual behavior offer a unique contribution to our understanding of HPV natural history. 

In these mid-adult cohort studies, reporting sex with recent new, or otherwise high-risk male sex partners (e.g., casual partners or partners with other concurrent partners) increased the risk of incident HPV detection [4,14,17]. Among women with recent new, or high-risk partners, the fraction of new HPV detection that could be attributed to one of these recent partners was estimated to be between 64 [17] and 82% [14], with the remainder likely due to re-detection of prior infection. It is important to note that these estimates that measure attributable risk in the “exposed” (i.e., those with recent new partners) are distinct from population attributable risk. The latter measure is dependent not just on the relative strength of new partners as a risk factor for new HPV detection, but also on the proportion of women in a study population who report new partners. Notably, in the Baltimore cohort, although incident HPV detection was 5.6-fold more likely in women reporting recent new partners than in women reporting no recent sexual activity (a strong relative risk), the fraction of all incident HPV detections attributable to recent partners was low (27%) because only 10% of women reported a new sex partner during study follow-up. Conversely, 72% of apparent new HPV detection in this cohort was attributed to a lifetime number of ≥5 male sex partners. In addition, the relative risk of detection associated with lifetime partners increased with age, further suggesting a relative shift from new acquisition to reactivation as the more likely source of new HPV detection in older women. In the Seattle cohort, recent sexual behaviors were also associated with increased risk of incident HPV detection, but only in the absence of serologic evidence of prior infection with the same type [4]. Compared to the Baltimore cohort, reports of new partners and other recent high-risk sexual behaviors were more common in the Seattle cohort; thus, the fraction of incident HPV detections attributable to recent sexual risk behavior was higher. A combination of sexual behavior and baseline serology data were used to create a composite variable to reflect risk categories for type-specific incident HPV detection: seronegative with no recent high-risk sexual behavior, seronegative with recent high-risk sexual behavior, and seropositive (irrespective of recent sexual behavior). With this approach, 40% of incident HPV in the Seattle cohort was attributed to probable new infection, due to a lack of serologic evidence of prior infection coupled with recent sexual risk behavior. On the other hand, 30% of incident detections were attributed to likely re-detection of prior infection due to the presence of natural antibodies to the same type. Of note, while the proportion of new detections attributable to prior infection remained constant with age, the proportion attributable to probable new infection was higher in women aged 30–39 years (48%) than in women aged 40–50 years (21%). Declining rates of new acquisition with age (as seen in the other mid-adult cohorts) combined with possible waning antibodies with age (such that a larger proportion of new detections due to prior infection could not be classified as such in the absence of natural antibodies) are likely explanations for these findings. 

Results from these three cohort studies indicate that, while new partners remain a strong risk factor for new HPV infection into mid-adulthood, the likelihood that newly detected HPV is due to new infection versus re-detection of prior infection declines with age. In these studies, it is also notable that recent sex with non-new or otherwise low-risk partners did not appreciably increase the risk for incident HPV detection compared to no recent sexual activity. This information may be reassuring for women in monogamous relationships who test HPV-positive in clinical settings. 

Natural history studies in older women using similar strategies for attribution of new HR-HPV detection to acquisition vs. recurrent detection, but with a longer duration than the Baltimore and Seattle studies, are required to better estimate whether there is similar or differential risk for cervical precancer or cancer. Since the studies cited above consistently show an increasing attributable fraction of new HPV detection due to prior infection relative to recent acquisition with age, precancer risk by age provides a reasonable surrogate for risk difference by the source of HPV infection. In a large US study of women receiving routine co-testing, the risk of CIN3+ in women with newly detected HR-HPV was largely similar across age groups [26], suggesting no difference in risk in women with newly acquired vs. recurrent detection of HR-HPV. From the perspective of clinical management of newly detected HR-HPV in screening, similar risks independent of the transition pathway to HPV detection as depicted in Figure 2 is reassuring since unequivocal differentiation of the transition path for any given woman is currently impractical. However, this does not obviate the need to develop a more complete understanding and differentiation of these natural history pathways, because while patients with transiently detected HR-HPV are reassured by a negative test as it relates to their immediate precancer risk, they remain concerned about how to protect themselves from re-infection.

## 6. Protection against Re-Infection: Role of Naturally Acquired Antibodies and Vaccine

A key area of controversy is whether serum antibodies from natural HPV infection protect against re-infection with the same HPV type. To date, studies addressing this issue have produced mixed results [27,28,29,30,31,32,33]. In a study of the control arm of a bivalent HPV vaccine trial, Safaeian and colleagues reported a 50% and 64% reduction in incidence of HPV16 and HPV18 detection, respectively, in women with the highest levels of HPV antibodies [27]. It should be noted, however, that HPV antibody titers are generally not bimodal, and selection of a cutpoint to define seropositives is somewhat arbitrary, often relying on statistical definitions of 3–5 standard deviations over the mean optical density (OD) of virginal (presumably HPV-negative) girls [32]. Thus, it is possible that a lack of protection associated with lower HPV antibody titers may be the result of misclassification of baseline HPV serostatus, rather than a lack of protection associated with lower antibody titers. Other studies suggest that antibodies may offer protection against type-specific re-infection that wanes with age [8,12]. In the placebo arm of the quadrivalent HPV vaccine trial in mid-adult women, protection against type-specific re-infection was observed for women aged 24–34 years, as demonstrated by a higher incidence of vaccine-type HPV in seronegative versus seropositive women (5.7 versus 1.0 per 100 person-years) [34]. In contrast, in women aged 35–45 years, the incidence of vaccine-type HPV was observed to be slightly higher in seropositive versus seronegative women (2.8 versus 2.1 per 100 person-years), suggesting a possible lack of protection from natural infection in older women. An important consideration, however, is that reactivation of previously acquired, latent HPV infection, rather than re-infection, may explain the apparent lack of protection against re-infection from natural antibodies in the older age group. In the Seattle mid-adult cohort, type-specific HPV incidence was also observed to be higher in seropositive versus seronegative women (with a 6-month cumulative incidence of 2.9% versus 1.2%, respectively), a trend observed both in 30–39 and 40–50 year old women. Furthermore, as noted above, recent sexual behaviors were unassociated with new HPV detection in the presence of serologic evidence of prior infection with the same type (suggesting a low likelihood of re-infection versus re-detection of prior infection) [4]. These results suggest that, in the presence of naturally acquired antibodies, reactivation or intermittent viral shedding of a previously acquired infection is a more probable source of new HPV detection than new acquisition. This theory is further supported by earlier studies showing that it is rare for an individual woman to be infected with more than one variant of a specific HPV genotype [35].

HPV vaccine trials in mid-adult populations (aged 26–45 years) show similar efficacy in women naïve for the vaccine types at the time of immunization (e.g., both DNA and seronegative) [36,37], suggesting that women at risk for new HPV exposures may benefit from HPV vaccination at any age (though we note that new partner acquisition declines precipitously with increasing age [21] and thus the value of routine immunization of mid-adult women may have little benefit at the population level). In contrast, women with currently detected vaccine-targeted DNA at the time of immunization are not protected from progression to CIN3+, nor do they control their infections more quickly compared with control arm women [38]. Previously infected control arm women who were seropositive but DNA-negative at the time of immunization (i.e., women with prior, but not current infection) had a lower risk of new vaccine-type detection compared with seronegative controls. Yet, the rate of new vaccine type detection in the seropositive vaccine arm participants was significantly lower than seropositive controls [36], suggesting the possibility that vaccination may reduce risk of reactivation of controlled infection. It has been shown that B-cell memory is substantially boosted in vaccinated women with prior exposure to vaccine types, and that neutralizing antibodies were elicited in all women with a single dose vaccine boost, but only in a limited number of women with natural memory B-cell-derived antibodies [39]. These data provides some biological plausibility for enhancement of immunologic control of previously acquired HPV infections to prevent, or reduce, the frequency or duration of reactivation (see future research needs below).

## 7. Rationale for Resolving Remaining Uncertainties

Why is it important to resolve the remaining uncertainties in our understanding of HPV natural history? From a clinical perspective, clarifying the unknowns has critical implications for patient psychosocial counseling. As women participating in cervical cancer screening accumulate HPV testing histories, there is a strong need for comprehensive, accurate, and reassuring information to guide clinician–patient interactions for complex, yet common scenarios such as non-consecutive HPV-positive results. While the increased risk of carcinogenic progression associated with persistent HPV detection is clear [3] and risk associated with newly detected HPV is similar across age [26], it remains uncertain whether an intermittent viral detection or reactivation history increases the risk of progression compared to a history of consistent negative HPV test results. Several health systems in the United States have accumulated over a decade of HPV co-testing data (e.g., Kaiser Permanente, Northern California, CA, USA) and are well suited to evaluate risk associated with decades of negative HPV testing vs. persistent detection vs. intermittent detection. A recent report from the POBASCAM study of HPV testing in the Netherlands showed a higher risk of new HPV detection and CIN3+ development in women with intermittent negative HPV tests in screening compared with women remaining persistently HPV-negative. Recurrent positive results following apparent clearance were most often due to the same type, suggesting reactivation rather than new infection [40].

From a public health perspective, accurate natural history models are necessary for developing evidence-based HPV vaccination and cervical cancer screening strategies for older populations of women. For example, while data from vaccine trials in mid-adult women suggest that the protective benefits of prophylactic HPV vaccines may extend beyond newly acquired infection—with clear efficacy demonstrated in the subgroups of women with baseline antibodies to vaccine-type HPV compared to seronegative women [36]—the underlying mechanism of protection is unclear. The mathematical models used to estimate vaccine effectiveness and cost-effectiveness in older populations of women are sensitive to assumptions regarding natural immunity, viral latency, and re-infection [8,33,41]. More accurate parameters are needed to inform the validity of bold strategies such as HPV-FASTER [42] that propose integrated screening, treatment, and vaccination programs in women up to 45–50 years of age. 

However, elucidating a more accurate picture of the natural history of HPV infection within women over time will require different approaches to study design and analysis. For example, Liu et al. reported on the temporal dynamics of type-specific HPV infection in women with bi-weekly sample collection over 4 months, and found that many types were repeatedly detected with very short duration (<2 weeks) over the course of the study [6]. Recurrent detection was not associated with sexual activity, but was associated with stage of the menstrual cycle [43] and microbiome transitions [44], suggesting a much more dynamic reactivation/control of virus in the lower genital tract than has been previously acknowledged. The evolving understanding of viral natural history when employing more frequent and intensive sampling designs has strong analogies with herpes simplex virus type 2 (HSV-2), where a paradigm of lifelong latency with infrequent reactivation was replaced with a model of very frequent bursts of viral reactivation and shedding, with local immunologic memory keeping the duration of these shedding episodes to a few hours (reviewed in [45]). Using data collected from HSV-2 seropositive women with multiple genital sample collections per day (up to every 6 h while awake), mathematical simulation models were generated to both explore new hypotheses about the mechanisms of HSV-2 reactivation/control cycles and the impact of interventions [46]. Similar studies are envisioned to provide critical evidence for the mechanisms of HPV recurrent detection, as well as the possible impact of vaccine and probiotic or immunologic interventions aimed at minimizing the frequency and/or duration of recurrent HPV detection. This could translate broadly in terms of public health impact on cervical precancer/cancer risk in adult women, as well as potentially reducing the newly recognized risk of HPV infection (and “clearance”) on increasing the susceptibility to HIV infection in populations with high co-infection rates.

Viewed from the lens of an infectious disease epidemiologist, the epidemiology of female genital HPV infection across the life span is likely more complex than previously appreciated. Evidence supporting an important transition pathway from HPV detection to non-detection, which represents virologic control or latency, rather than viral clearance or eradication, is rapidly accumulating. As women across the world begin to accumulate their own personal HPV testing histories, understanding the risks of precancer/cancer incidence associated with intermittently positive vs. repeatedly negative tests results becomes imperative for setting rational clinical management guidelines for screening intervals and safe exiting from screening in older women. In addition, the notion of prophylactic vaccination in older, less sexually active women is gaining in popularity. However, the individual- and population-level benefits of vaccination in women well past their sexual debut is dependent on understanding the proportion of new HPV detection resulting from recent acquisition vs. recurrent detection/reactivation, as well as the potential role for HPV vaccines in preventing or controlling reactivated infection. In other words, the time to act on improving our understanding of the causes and consequences of HPV latency and prevention of reactivation is now.
